# Transcranial Direct Current Stimulation of motor cortex enhances running performance

**DOI:** 10.1371/journal.pone.0211902

**Published:** 2019-02-22

**Authors:** Seung-Bo Park, Dong Jun Sung, Bokyung Kim, SoJung Kim, Joung-Kyue Han

**Affiliations:** 1 Department of Physiology, KU Open Innovation Center, Research Institute of Medical Science, Konkuk University School of Medicine, Chungju, Chungbuk, South Korea; 2 Division of Sport and Health Science, College of Biomedical and Health Science, Konkuk University, Chungju, Chungbuk, South Korea; 3 Department of Physical Therapy and Kinesiology, Zuckerberg, College of Health Sciences, University of Massachusetts, Lowell, Massachusetts, United States of America; 4 College of Sport Sciences, Chung-Ang University, Anseong, Gyoenggi, South Korea; University of Ottawa, CANADA

## Abstract

Transcranial direct current stimulation (tDCS) is a technique used to modulate neuronal excitability through non-invasive brain stimulation that can enhance exercise performance. We hypothesize that tDCS would improve submaximal running time to exhaustion (TTE) and delay the increase in the rating of perceived exertion (RPE) over time. We also hypothesize that tDCS would not lead to difference in cardiorespiratory responses. We employed a randomized, single-blinded, and counterbalanced design in which 10 trained men participated. After receiving either 20 min of 1.98 mA anodal tDCS applied over the primary motor cortex (M1) or sham-operated control on separate days, participants completed a constant-load test involving running at a speed equivalent to 80% of their own maximum oxygen consumption (VO_2_max). During this constant-load test, RPE, heart rate (HR), VO_2_, pulmonary ventilation (VE), respiratory exchange ratio (RER), and ventilatory threshold (VT) were continuously monitored. TTE was recorded at the end of the test. TTEs were significantly longer in the tDCS than in the sham conditions (21.18 ± 7.13 min; 18.44 ± 6.32 min; *p* = 0.011). For TTE, no significant differences were found in RPE between conditions at isotime. In addition, no significant differences in HR, VO_2_, VE, RER, and VT were found during TTE between the two stimulation conditions at any time point. These results indicate that the application of tDCS does not induce a change of the exercise performance-related index; however, it can affect the increase of the exercise duration due to the stimuli in the M1 area.

## Introduction

Supraspinal fatigue refers to a failure of the primary motor cortex (M1) to generate output, and when combined with the peripheral fatigue mechanism, it is involved in muscle fatigue [1]. Transcranial direct current stimulation (tDCS) interventions to increase M1 excitability may improve endurance performance by increasing the motor output of the M1 and delaying supraspinal fatigue [2, 3]. Hence, tDCS reduces pain and instigates motor unit recruitment by sending a weak electrical current to the scalp and modifying neuronal excitability to modulate brain functions [4, 5].

During tDCS application, anodal stimulation enhances cortical activation, whereas cathodal stimulation inhibits it [6, 7]. Furthermore, tDCS alters the resting membrane potential of neurons in a specific area and induces brain plasticity [8, 9]. A pharmacological study has shown that a tDCS-related effect increased cortical excitability by modifying the activation of N-methyl-D-aspartic acid receptors [10]. A few studies have recently investigated the acute effect of tDCS on endurance performance [11, 12]. Vitro-Costa et al. [13] showed that anodal stimulation in M1 improved cycling time to exhaustion (TTE) in recreationally active men.

In a study conducted by Okano et al. [14], the average TTE of trained cyclists increased when anodal stimulation was applied to the left temporal cortex (T3), while the average the rating of perceived exertion (RPE) relatively decreased. The magnitude of central motor command derived from activity in the motor/premotor brain areas is associated with changes in RPE [15, 16]. Therefore, if M1 excitability is increased after tDCS administration, less input is needed to induce the amount of output required to reinforce the muscle, which indicates that a lower RPE is required for a given force or power [11]. In a study by Vitor-Costa et al. [13], RPE did not change, despite the improvement in TTE. This finding indicated that increased M1 excitability makes the individual feel that the exercise of a given intensity is easier than it would otherwise be perceived [3, 17].

During exercise, afferent feedback is a major factor that can modulate cardiovascular responses [18]. Amann et al. [18, 19] have shown that afferent feedback plays an important role in cardiovascular regulation. However, they did not explain how afferent responses contributed to endurance performance. Recently, a few studies have shown that M1 excitability is not related to cardiovascular regulation [13, 20, 21]. Thus, it is necessary to clarify the association of endurance performance with afferent feedback and cardiovascular system. It has been suggested that M1 has an important influence on exercise performance, the perception of effort, and cardiovascular responses [13].

However, whether endurance performance is affected by tDCS during submaximal running exercise remains unclear. Furthermore, the whole-body dynamic exercise investigated in this context has thus far been limited to cycling, and no studies have been conducted on running. Therefore, identifying the effects of tDCS in the cerebral cortex could play an important role in improving performance as a strategy to elicit the potential of the human body [22]. Therefore, the purpose of this study was to examine whether the ergogenic effects of tDCS would improve endurance performance. We hypothesized that tDCS would increase submaximal running time to exhaustion (TTE), delay the increase in RPE over time, and show no changes in cardiorespiratory responses.

## Materials and methods

### Participants

Twelve trained male participants volunteered for this study. All participants were evaluated by medical examination and physical activity readiness questionnaire (PAR-Q). Participants were instructed to refrain from consuming analgesic medications (6 h), alcohol (48 h), and caffeine (6 h), as well as to refrain from intense exercise (48 h) prior to every visit. In addition, they were informed of the overall experimental procedure and risk factors related to the measurements, but not of the study objectives or hypotheses. Written informed consent was obtained from each participant. The research protocol for this study was approved by the Chung-Ang University Institutional Review Board (NO. 1041078–201706–HRZZ–116–01).

### Study design

Each participant visited the laboratory a total of five times. The study was designed as a single-blinded, randomized, counter-balanced study. Tests were scheduled at the same time of day and laboratory conditions were kept stable (temperature 22 ± 0.5°C, humidity 47 ± 4%) for the duration of the study. All trials for each participant were completed within 3 weeks. All tests were separated by a 48–72 h recovery period in which participants refrained from any normal training activities.

The purpose of the first visit was to increase the validity of measurements by familiarizing participants with all measurement procedures. The VO_2_max test was conducted at incremental loads, and gas analysis exercise duration evaluations were all performed during the second visit. The purpose of the third visit was for participants to become familiar with their running speed and time. Visits *4* and *5* were single blinded and randomized in terms of the stimulation condition (either tDCS or sham-operated control). At each visit, the treatment corresponding to the assigned condition was provided, and a constant-load test was then performed.

#### VO_2_max test

Participants completed a warm-up treadmill run and stretching for 15 min (10 min at 8–11 km/h and stretching for 5 min). During the VO_2_max test, running began at a speed of 8 km/h and continued as the running speed was increased by 1 km/h every 1 min, until the participant was unable to continue the test. VO_2_max was considered as the attainment of at least two of the following criteria: 1) HRmax (bpm) ≥ 90% of the age-predicted maximum (calculated as 220 –age); 2) RER > 1.15; 3) RPE ≥ 17; and 4) plateau in VO_2_ ≤ 150 mL/min [23]. During the VO_2_max test and constant-load tests, cardiorespiratory variables were measured breath-by-breath using an automated gas analyzer (Metalyzer 3b, Cortex, Leipzig, Germany), and participants wore a face mask of an appropriate size (S or M size). Prior to downloading of information to a personal computer, the gas concentrations and respiratory volume were first stored in on-board memory. Following the recommendations of the manufacturer, the metabolism analyzer was turned on for more than 20 min and then calibrated prior to each use. In reference to the suggested gas composition (16% O_2_, 5% CO_2_, balance N_2_: ± 0.02% absolute, Hong Kong Specialty Gases, Hong Kong, China), the gas was then analyzed and calibrated against ambient air. Additionally, a standardized 3 L syringe (5530 series, Hans Rudolph, Inc. Shawnee Mission, KS, USA) was used for volume calibration. The transmitter strap of the HR monitor was fixed to each subject’s chest. HR was continuously monitored by a Bluetooth device during all tests (Polar H7, Polar Electro OY, Kempele, Finland). HR data and all cardiorespiratory variables were collected by every 5 s.

#### Constant-load test

**Fig 1:**
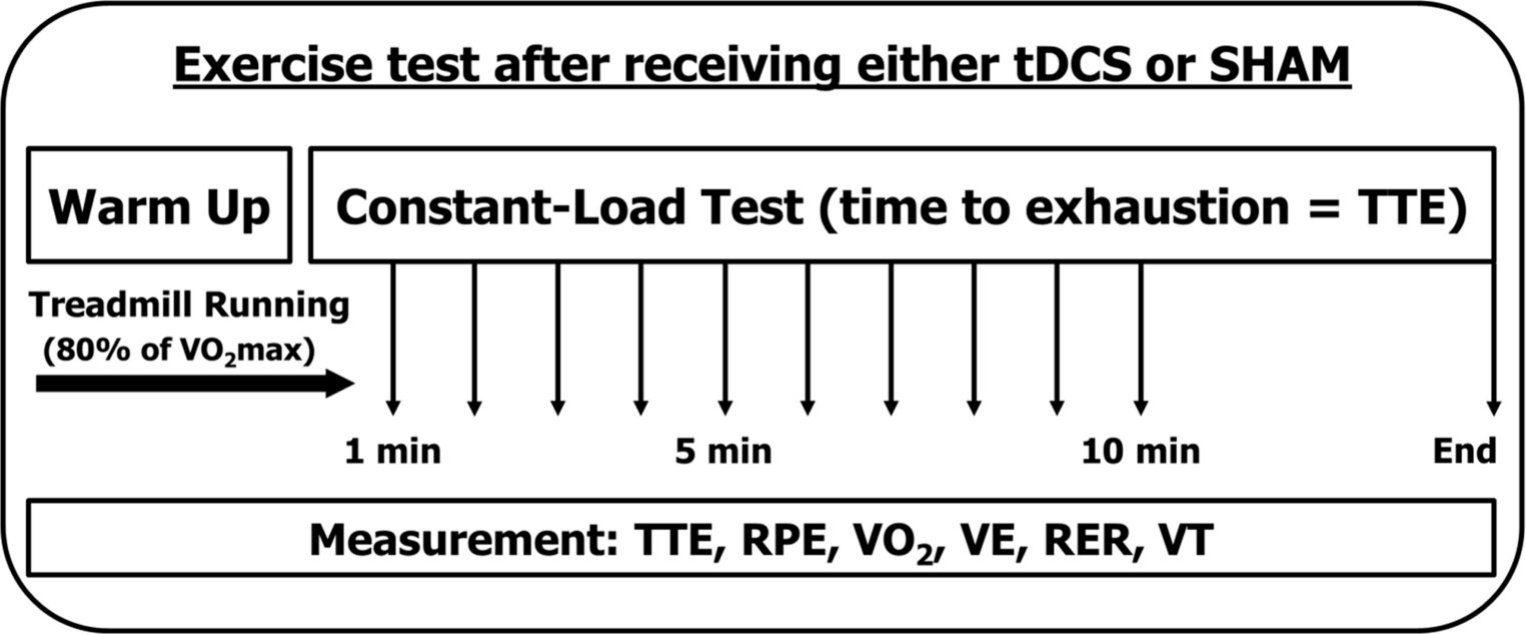
Constant-load test procedure.

During the next two visits, the participants completed a constant-load test. The TTE was measured while they ran at a speed equivalent to 80% of their own VO_2_max obtained using the American College of Sports Medicine (ACSM) running equation [24], until they could not continue running any longer. During the constant-load tests, rating of perceived exertion (RPE), heart rate (HR), oxygen consumption (VO_2_), pulmonary ventilation (VE), and respiratory exchange ratio (RER) were recorded at the end of each minute of the tests. In RPE, participants were instructed to focus on exercise-induced fatigue during constant running. An isotime of 10 min plus the final min for TTE were used to include all participants’ data in the subsequent analyses (Fig 1). Ventilatory threshold (VT) point was measured using the ventilatory equivalents method [25]. VT was determined at the point at which the VE/VO_2_ curve increased while the VE/VCO_2_ curve decreased or stayed the same. Additionally, participants were not provided with any information about their running distance or time.

#### tDCS procedure

For tDCS, a Halo Neurostimulation (Halo sports, Halo Neuroscience, San Francisco, CA, USA) device was used. The size of the electrodes affixed to the scalp was 28 cm (6.4 cm × 4.4 cm). The anodal electrode was positioned on CZ (midline central) the ‘vertex’ top of the head, and the cathodal electrodes were placed on C5 and C6, following the international 10–20 EEG System [26]. This electrode position is highly correlated with functional magnetic resonance imaging (fMRI) [27]. The current of the anodal tDCS was set at 1.98 mA, and the duration was set at 20 min. In the sham condition, the current was first run for 30 s, after which it was terminated. Participants were blinded as to the polarity of the stimulation condition (either tDCS or sham).

In this study, participants first rested, sitting in a chair for 15 min and then wore a headset with a tDCS device for 20 min while they received the corresponding treatment, during which they completed a warm-up (running and stretching) for 15 min and then a five-min rest period. Afterward, the tDCS headset was removed, and a constant-load test was performed.

### Statistical analysis

Data were processed and analyzed using SPSS-PC 22.0 software (SPSS, Chicago IL, USA). The mean and standard deviation (SD) for all variables were computed. The normality of the data distribution was assessed using the Shapiro–Wilk test, and the sphericity was examined using Mauchley’s test. Post-hoc statistically significant effects were determined using Wilcoxon signed-rank test. Effect sizes (ES) (*r* = Z/√*N*) were calculated for non-parametric tests [28]. The standard values of *r* for small, medium, and large sizes were 0.1, 0.3, 0.5, respectively [29]. Analyses of RPE, HR, VO_2_, VE, and RER at different moments during TTE were performed using repeated measures ANOVA. Additionally, TTE and VT were assessed using paired sample *t*-tests. Statistical significance was set at *p* < 0.05. ES were calculated using Cohen’s *d* for parametric tests and interpreted using the recommendations proposed by Hopkins [30]: < 0.2 trivial, < 0.6 small, < 1.2 moderate, < 2.0 large, < 4.0 very large, and 4.0 nearly perfect.

## Results

Regarding general characteristics of participants, age, height, weight, % body fat, maximal oxygen consumption, maximal heart rate, and time to exhaustion at VO2max were 27.40 ± 2.37 yrs, 174.13 ± 3.61 cm, 71.53 ± 7.47 kg, 12.29 ± 3.38%, 54.03 ± 5.03 ml/kg/min, 197.60 ± 5.8 bpm, and 8.97 ± 1.17 min, respectively.

**Fig 2:**
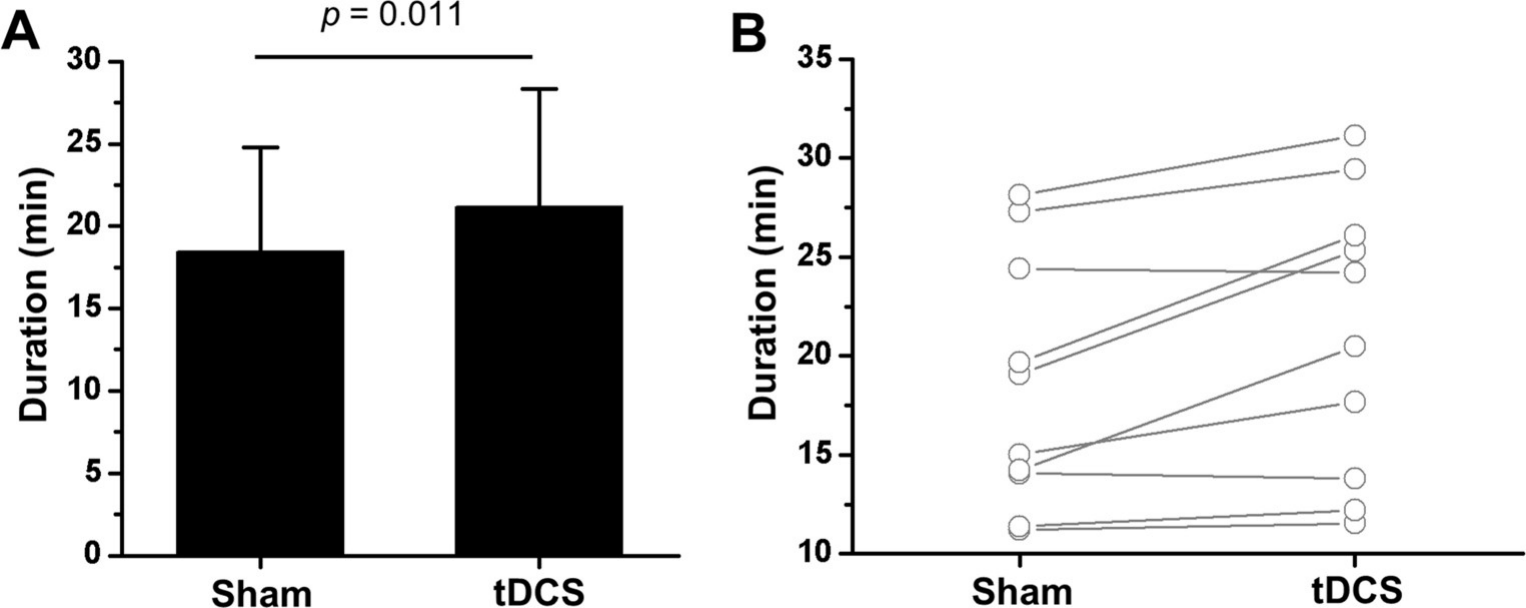
Effects of tDCS application on the TTE. **A**, TTE under different experimental conditions. **B**, Change in individual TTE. The *p* value was calculated by paired sample t-test.

We examined the effects of tDCS application on TTE under submaximal treadmill running. As shown in Fig 2A and 2B, TTE were significantly longer in the tDCS application than in the sham-controlled condition (*t* = -3.213, *p* = 0.011, *d* = 0.407). In addition, TTE of 7 participants was increased by tDCS application. These results indicate that the application of tDCS did not induce a change in the exercise performance-related index; however, it could increase the exercise duration due to stimuli introduced to M1 area.

**Fig 3:**
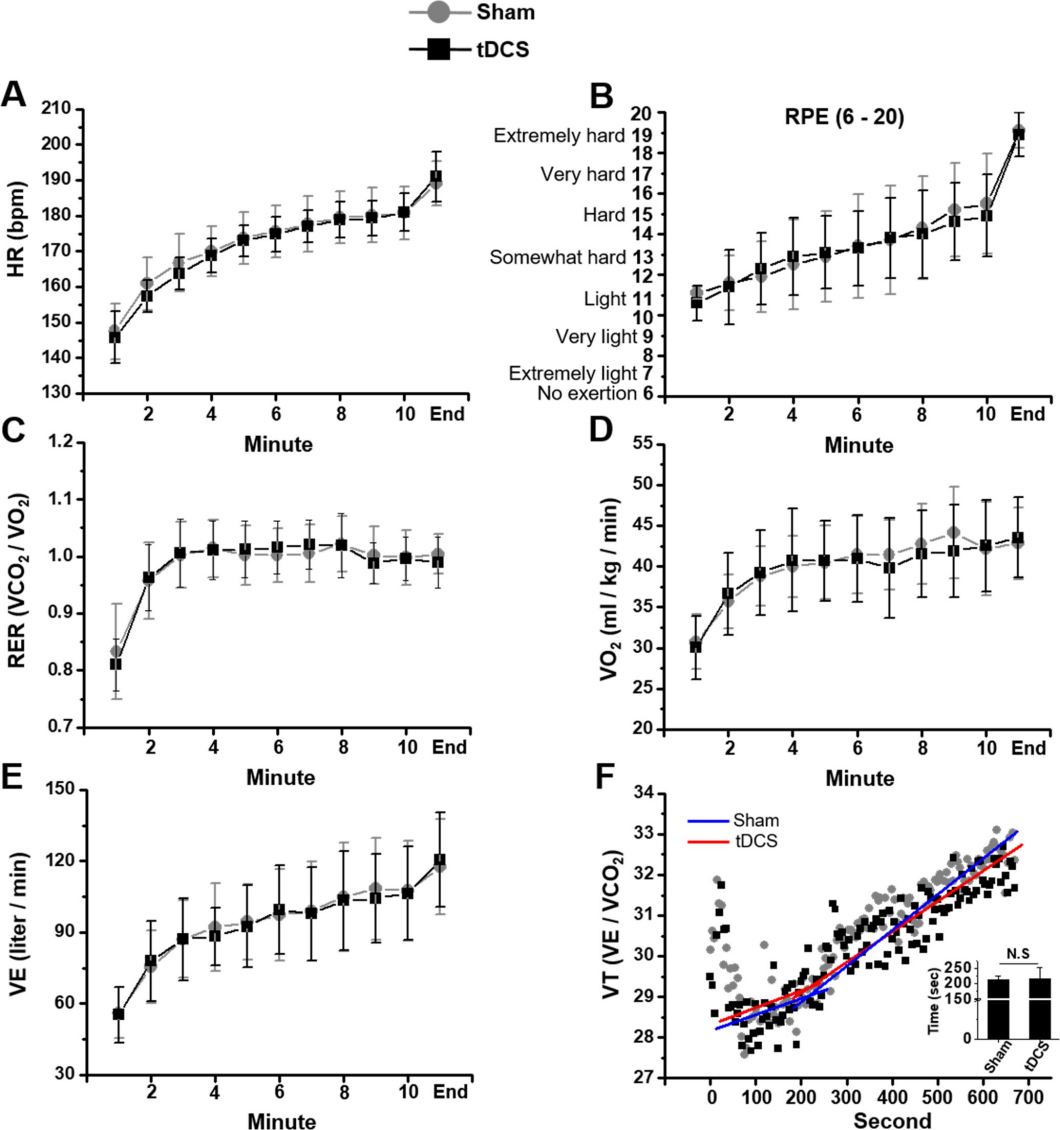
Effects of tDCS application on the treadmill running performance. **A**, HR in the sham and tDCS application. **B**, RPE in the sham and tDCS applications. **C**, RER in the sham and tDCS application. **D**, VO_2_ in the sham and tDCS applications. **E**, VE in the sham and tDCS applications. **F,** Representative trace recording of VT in the sham and tDCS applications and VT time (insert graph). N.S, not significant difference.

As shown Fig 3A, HR was not significantly different between sham and tDCS application (*p* = 0.610, *d* = 0.193). For observation of participant’s subjective intensity, we tested the RPE during treadmill running in the absence and presence of 2 mA current by tDCS (Fig 3B). There were no differences in the values for RPE between measurement times according to tDCS applied condition.

Despite the application of tDCS, RER did not show significant difference compared to sham (Fig 3C, Wilks’ Lambda = 0.472, *p* = 0.501, *d* = 0.024). Similar to RER, according to the tDCS application, VO_2_ (Wilks’ Lambda = 0.314, *p* = 0.161, *d* = 0.047) and VE (Wilks’ Lambda = 0.399, *p* = 0.328, *d* = 0.036) did not show any significant difference at the same measurement time (Fig 3C and 3E).

We observed a VT known as an index for exercise intensity setting (Fig 3F). The VT of sham and tDCS were 211.50 ± 13.75 and 216.50 ± 35.74, respectively, and there was no significant difference (*t* = -0.052, *p* = 0.959, *d* = 0.008).

## Discussion

The effects of tDCS on submaximal running performance have not been previously reported. Our main finding is that the M1 montage improved TTE when running at 80% intensity, but no change was found in cardiorespiratory responses.

In this study, TTE was improved in comparison with the sham condition after tDCS treatment, which indicates that tDCS induces ergogenic effects during running. These results are consistent with those of previous studies, showing an analgesic effect during cycling after a tDCS intervention in recreationally active men [14, 31, 32]. "Pain" plays an essential role in exercise tolerance due to its afferent feedback [11]. Pain experienced during high intensity exercise is caused by various exercise-induced metabolites that can be detected by surrounding muscle nociceptors categorized as group III and IV muscle afferents [20]. The signal is then detected as pain in frontal lobe regions [11]. Since conscious awareness of oneself is a significant factor in deciding one’s pacing strategy during exercise, exercise-induced pain may help an individual better understand the relevant tension on the body. This can help the individual make an informed decision to modulate exercise intensity [33]. A representative study by Okano et al. [14] reported an increase in TTE after tDCS treatment in 10 cyclists, suggesting that a montage with the anode over the left temporal cortex induces a pleasant sense of reduced effort and exercise-related discomfort in the early stages of the task.

In contrast, some studies reported no TTE improvement observed with tDCS [34]. Angius et al. [20] have used a montage with the anode targeted to the left motor cortex and the cathode targeted to the dorsolateral right prefrontal cortex (DLPFC) [35, 36, 37]. This montage increased excitability in M1 while the cathode reduced excitability in the DLPFC [20]. tDCS intervention of the DLPFC is negatively correlated with perceived pain [38]. It can reduce emotional response to pain [39, 40]. Therefore, M1 activation can be nullified by cathodal DLPFC stimulation [20]. In the present study, however, we believed that anodal stimulation did not affect C5 or C6 position related to the temporal lobe (i.e., insula and thalamus). This is supported by the study of Vitor-Costa et al. [13], showing that TTE is improved in a group receiving anodal stimulation of M1 alone. In addition, Angius et al. [21] have shown an improvement in TTE by applying an anodal stimulus to M1 and attaching the cathodal stimulus to a non-brain area (contralateral shoulder). However, there was no change seen in endurance performance when the cathodal electrode was applied to the prefrontal region. Therefore, positioning cathodes at C5 and C6 could play a role in TTE improvement.

Our study found that RPE was unchanged whereas other studies found that RPE was decreased. These results demonstrate that an RPE decrease after tDCS administration can activate M1, increase central motor command, and show a relatively lower perception of efforts. Such RPE change is related to various activities across different regions in the motor cortex, including premotor and M1 [15, 16]. Theoretically, anodal tDCS administration can decrease discomfort levels and positively affect RPE subsequently, thus improving exercise tolerance [11]. In this context, it has been reported that anodal stimulation in the left dlPFC can positively affect the control of emotions [41, 42, 43] and decrease perceived discomfort levels [44]. Unlike the dorsolateral prefrontal cortex (DLPFC) which is directly associated with emotions such as discomfort levels, the montage used in this study applies an anodal stimulation to CZ (M1) which is not generally related to emotion control. Nevertheless, as the frontal cortex is connected through the cortico-cortical neural networks [45, 46], tDCS stimulation in this study might have affected not only the M1 region in the cortex, but also subcortical structures. In addition, the perception of efforts might have been different owing to participants’ motivation and emotions under a single-blinding design. An interoceptive model which considers various factors can be used to explain this study’s results. According to the interoceptive model, various factors including afferent feedback, sensory, emotion, and motivation collectively contribute to central fatigue based on physiological state of the whole body [47]. This model is an integrative model that supplements existing neurochemical and psychophysiological theories. However, most recent studies suggest that anodal tDCS increases TTE by decreasing RPE. Thus, further study is needed on this subject matter.

Previous studies have reported that the afferent responses from peripheral muscle nociceptors (group III and IV muscle afferents) regulate cardiorespiratory responses during exercise [18, 19]. Okano et al. [14] reported that tDCS over the temporal cortex increases the heart rate variability (HRV), which is modulated by the autonomic nervous system (ANS) activity during incremental exercise testing. In addition, Montenegro et al. [48] showed that tDCS at the left temporal lobe targets the ANS control areas appreciably, leading to increased HRV in healthy subjects. Taken together, these results suggest that tDCS could increase ANS activity during exercise. In this study, however, we found no significant difference in SNS/PNS balance. This might be attributable to exercise intensity. More specifically, in this study, we performed a constant-load test at submaximal intensity and accordingly produced results with reduced parasympathetic activity. Okano et al. [49] have reported no significant difference in SNS/PNS balance among 13 young adult sedentary men who performed exercise at a constant-load test of 80% HRmax. In addition, Anguas et al. [20] have found no effect of exercise intensity on cardiovascular responses, indicating that exercise intensity could affect SNS/PNS balance. However, whether cardiorespiratory responses are affected by tDCS during exercise remains unclear. Vitor-Costa et al. [13] reported that when 2 mA stimulation was applied to 11 recreationally active men for 13 min and their HR and HRmax were compared according to exercise time, they showed no significant differences compared to the sham group.

Similar to these previous studies, the present study also did not find any significant changes in cardiorespiratory response (HR, VO_2_, VE, RER, and VT) at any time point after tDCS treatment in comparison to the sham conditions. Consequently, as in previous studies, the findings of the present study support the theory that tDCS has no effect on the physiological variables associated with cardiorespiratory response (HR, VO_2_, VE, RER, and VT) and suggest that communication between the central nervous system and motor units is regulated solely by afferent responses. Although the tDCS montage used in each study may vary, the findings reported to date suggest that tDCS intervention has no effect on cardiorespiratory responses (HR, VO_2_, VE, RER, and VT) during exercise.

Several limitations of the present study should be noted. Although electric stimulation was applied specifically to target sites during tDCS treatment, it may have influenced other areas in the vast cortex. In other words, we have limitations in precisely screening sites that are stimulated by tDCS. However, in the present study, we believe that M1 is stimulated by tDCS according to various evidence. Jurcak et al. [27] have proposed that tDCS is highly correlated with EEG and fMRI based on 10–20 EEG. Meinzer et al. [50] have shown that the activation of M1 is significantly increased compared to sham as a result of confirming tDCS stimulation site by fMRI. The result of the present study can be considered to be mainly due to M1 stimulation. Although the tDCS device could be used in a double-blinded design, the present study had a single-blinded design.

## Conclusions

When tDCS was applied to the M1, it improves the TTE during submaximal running. However, tDCS intervention has no effect on cardiorespiratory responses. Future studies establishing the specific mechanisms between an analgesic effect and endurance performance are necessary. Additionally, while the acute effects of tDCS intervention have been validated, further studies are needed to determine the long-term effects on training following this intervention.

## Supporting information

S1 Table Results the cardiorespiratory capacities of each participant in the present study. (DOCX)
