# Supplementary material for: Transcranial Direct Current Stimulation of motor cortex enhances running performance
Source: PLoS One. 2019 Feb 22;14(2):e0211902. doi: 10.1371/journal.pone.0211902 (PMC6386265; doi:10.1371/journal.pone.0211902)
Supplement: S1 Table — (DOCX) [file pone.0211902.s001.docx]

| **S1 Table**. Results the cardiorespiratory capacities of each participant in the present study | | | | |
| --- | --- | --- | --- | --- |
| **Participants** | **VO_2_max (ml/kg/min)** | **HRmax (Beats/min)** | **VO_2_max-test Duration(min)** | **Constant-load speed(km/h)** |
| 1 | 55.77 | 199 | 9.37 | 12.3 |
| 2 | 56.46 | 206 | 10.37 | 12.3 |
| 3 | 50.49 | 197 | 8.9 | 10.9 |
| 4 | 56.67 | 200 | 8.77 | 12.6 |
| 5 | 56.42 | 199 | 8.02 | 12.3 |
| 6 | 46.07 | 204 | 7.52 | 9.9 |
| 7 | 57.01 | 185 | 10.42 | 12.6 |
| 8 | 61.42 | 194 | 10.13 | 13.5 |
| 9 | 45.87 | 198 | 7.42 | 9.9 |
| 10 | 54.11 | 194 | 8.13 | 11.9 |
